# Supplementary material for: Impact of the COVID-19 pandemic on early career dementia researchers: A global online survey
Source: PLoS One. 2022 Nov 9;17(11):e0277470. doi: 10.1371/journal.pone.0277470 (PMC9645634; doi:10.1371/journal.pone.0277470)
Supplement: S1 File — (DOCX) [file pone.0277470.s001.docx]

**File 1.** A list of dementia-specific resources for Early Career Researchers collected by the authors.

Note: This list does not aim to be exhaustive.

**WORLDWIDE**

- World Young Leaders in Dementia: <https://wyldementia.org>
  - Workshops
  - Collaborations
  - Networking
- Dementia Researcher: <https://www.dementiaresearcher.nihr.ac.uk>
  - Podcasts, (guest) blogs, and videos
  - Training and events
  - Career information

**EUROPE**

- INTERDEM Academy: <https://interdem.org/?page_id=4636>
  - Masterclasses, Academy Café (online)
  - Fellowships
  - Publication Award
  - Networking
- Alzheimer Europe Conference: <https://www.alzheimer-europe.org>
  - Award (Twitter “influencer”)
  - Training (Master classes in collaboration with INTERDEM Academy)
  - Networking

**ASIA**

- ASAD (Asian Society Against Dementia) [www.asaindementia.org](http://www.asaindementia.org)
  - ASAD Conference
  - Memberships privileges: access to information on Asian colleagues with shared interest and potential collaborative projects
  - Networking
  - Training
- ERIA (Economic Research Institute for ASEAN and East Aisa) <https://www.eria.org/>
  - Webinar: <https://www.eria.org/events/international-webinar-building-dementia-friendly-communities-in-asia-pacific/>
  - Networking
- ADI Asia Pacific Regional Conference
  - Networking

**NORTH AMERICA**

- International Society to Advance Alzheimer's Research and Treatment (ISTAART) and Professional Interest Area to Elevate Early career Researchers (PEERs PIA): <https://action.alz.org/PersonifyEbusiness/Default.aspx?TabID=1739>
  - Webinars
  - Alzheimer’s Association International Conference (AAIC) ECR sessions
  - Volunteer and Leadership Opportunities
  - Networking
  - Overall ISTAART and Professional Interest Area publication and poster presentation awards
  - Career information and job postings through the ISTAART Career Center (<https://alz-jobs.careerwebsite.com/jobs/>)

**SOUTH/ CENTRAL AMERICA**

- Latin American Training Program (LATP; Society for Neuroscience)
  - Year-long online training program
  - Webinars and online discussions
  - ECR’s from Latin America and Caribbean are eligible
  - Accepted applicants participate in the online program. The 15 top candidates are selected to participate in a three-week long in-person course
- CALDO – Connecting ECR’s with top Canadian universities
  - Offers the opportunity for Latin American students to pursue their higher education studies in Canada, with Neuroscience as one of the priority areas
- LAC-CD – Latin American and Caribbean Consortium on Dementia
  - Networking
  - Webinars
  - Opportunities to participate in collaborative efforts
- Fulbright Scholar Program
  - Opportunities for ECRs and faculty member around the world to spend a year in a institution in the USA, among other programs

**AUSTRALIA**

- Australian Dementia Research Forum: <https://www.australiandementiaresearchforum2022.org.au/>
  - National conference
  - Networking
  - Webinar
- Australian Dementia Network Early and Mid-Career Researchers Accelerator Group: <https://www.australiandementianetwork.org.au/researcher/>
  - Sponsorship program
  - Workshops & Webinars
  - Training
  - Grant opportunities: <https://protect-au.mimecast.com/s/qqcVCD1vmxc5pXVKckXp-6?domain=docs.google.com>
- Australian Association of Gerontology Student and Early Career Group (SECG): <https://www.aag.asn.au/about-us/student-and-early-career-group-secg>
  - Clinical ECR focus
  - Networking
  - Collaboration
  - Mentorship
  - Conference
- Step Up For Dementia Research: <https://www.stepupfordementiaresearch.org.au/>
  - Facilitate recruitment
- Wicking Dementia Centre <https://www.utas.edu.au/wicking>
  - Online global education and interdisciplinary research
- The RAND Corporation <https://www.rand.org/about/glance.html>

**AFRICA**

- MHIN Africa (The Mental Health Innovation Network) <https://www.mhinnovation.net/collaborations/mhin-africa>
  - Database of mental health innovations in Africa
  - Resources developed by and for MHIN Africa members
  - Networking
  - SUCCEED Africa: SUpport, Comprehensive Care and EmpowErment for people with psychosocial Disability in Africa
- ‘TReND in Africa’ (Teaching And Research In Natural Sciences For Development In Africa) <https://trendinafrica.org/>
  - Funding & running of training courses across Africa on diverse emerging area skills including dementia research
  - Volunteering of Academic Researchers
  - Equipment donations
  - Community outreach programmes
  - Advocacy for African Science Policy Advancement
- IDSA Foundation <https://idsafoundation.org/alz-research-grant/>
  - Research Grants for Microbial Pathogenesis in Alzheimer’s disease.
- SciComNigeria (Science Communication Hub Nigeria) <https://www.scicomnigeria.org/>
  - Webinars & Podcasts on Dementia & other Research areas
  - Curation of database for Nigerian scientists across dementia and other science research disciplines
  - Annual National Life Sciences Competition (NLSC)
  - Public Communication of Science & technology to dispel misconceptions
  - Promotion of Collaboration between Nigerian researchers and journalists
  - Public engagement between aspiring scientists, journalists, educators and policymakers
  - Mentorship of students and junior researchers across Nigeria & Africa
- STIAS: Stellenbosch Institute for Advanced Study <https://stias.ac.za/fellows/projects/dementia-a-growing-problem-in-south-africa-and-the-world/>
  - Health Research
  - Fellowships
- Health Think Analytics <https://healththink.org/about-us/>
  - Collation, review, analysis and curation of African health data
- STRiDE: Strengthening responses to dementia in developing countries <https://stride-dementia.org/about-the-project/>
  - Understanding the impacts of dementia in various cultural, social and economic contexts in order to support development, financing, planning, implementation and evaluation of National Dementia Plans.
- Alzheimer's Disease International (ADI) <https://www.alzint.org/resource/dementia-in-sub-saharan-africa/>
  - Capacity building
  - Policy
  - Research
  - Partnerships
  - Accreditation
  - ADI Conference
- NIH Forgaty International Center <https://www.fic.nih.gov/Pages/Default.aspx>
  - Global Health Research News
  - Global Health Matters Newsletter
  - Conferences & Workshops
  - Webcasts & Videos
  - Publications
  - Fogarty Trainee Profiles
