# Supplementary material for: Impact of the COVID-19 pandemic on early career dementia researchers: A global online survey
Source: PLoS One. 2022 Nov 9;17(11):e0277470. doi: 10.1371/journal.pone.0277470 (PMC9645634; doi:10.1371/journal.pone.0277470)
Supplement: Attachment — Submitted filename: Response_to_reviewer_R1.docx [file pone.0277470.s002.docx]

Dear editor,

We appreciate the opportunity to revise our manuscript with your and the reviewer’s input. We hope to have incorporated all remarks well and answered all questions sufficiently. Below, we elaborate on each point individually.

With kind regards on behalf of all co-authors,

Dr Sara Laureen Bartels

**Journal Requirements:**

When submitting your revision, we need you to address these additional requirements.

1. Please ensure that your manuscript meets PLOS ONE's style requirements, including those for file naming. The PLOS ONE style templates can be found at
   <https://journals.plos.org/plosone/s/file?id=wjVg/PLOSOne_formatting_sample_main_body.pdf> and 
   <https://journals.plos.org/plosone/s/file?id=ba62/PLOSOne_formatting_sample_title_authors_affiliations.pdf>

>>> The supportive information file name was adjusted and all files should be in the correct format now.

2. Please amend your current ethics statement to address the following concerns:
a) Did participants provide their written or verbal informed consent to participate in this study?
b) If consent was verbal, please explain i) why written consent was not obtained, ii) how you documented participant consent, and iii) whether the ethics committees/IRB approved this consent procedure.

>>> On page 7, the informed consent procedure was further elaborated on (line 137-141): *“Via the online platform, participants read the study information and data use regulations, and then confirmed eligibility and provided written informed consent by ticking a box (“I agree”). A name or contact information was not collected. If participants did not confirm eligibility, or did not provide informed consent (“I disagree”), participation ended without collecting any information.”*

3. We note that the grant information you provided in the ‘Funding Information’ and ‘Financial Disclosure’ sections do not match.

When you resubmit, please ensure that you provide the correct grant numbers for the awards you received for your study in the ‘Funding Information’ section.

>>> This was checked in the platform, and the funding information in the manuscript were updated.

4. Thank you for stating the following financial disclosure: 
 "No funding was received for this work specifically. CES was funded by grant T32AG055381 from the National Institute on Aging of the United States National Institutes of Health. AS is funded by the National Institute for Health Research through the NIHR University College London Hospitals Biomedical Research Centre."

Please state what role the funders took in the study.  If the funders had no role, please state: "The funders had no role in study design, data collection and analysis, decision to publish, or preparation of the manuscript." 
If this statement is not correct you must amend it as needed. 
Please include this amended Role of Funder statement in your cover letter; we will change the online submission form on your behalf.

>>> The statement was included in the “Funding” section.

5. We noted in your submission details that a portion of your manuscript may have been presented or published elsewhere. "A summary report of the complete survey (raw data, non peer-reviewed) is also published under Smith A, Shaaban CE, Bartels SL, Welikovitch L, Brum W, Folarin R. Listening to Early Career Researchers [Report]. 2022 [Available from: <https://www.dementiaresearcher.nihr.ac.uk/survey.>" Please clarify whether this [conference proceeding or publication] was peer-reviewed and formally published. If this work was previously peer-reviewed and published, in the cover letter please provide the reason that this work does not constitute dual publication and should be included in the current manuscript.

>>> The published report is not peer-reviewed and includes descriptive information and graphs, without analysis or discussion of results. We now clarified this in the manuscript (page 7): *The survey created in English using www.surveymonkey.com, and covered several topics (e.g., experiences, job and workplace, conference attendance, publishing, moving countries, leaving academia), including the impact of COVID-19. The non-peer reviewed report with descriptive information can be accessed elsewhere (14).*

6. In your Data Availability statement, you have not specified where the minimal data set underlying the results described in your manuscript can be found. PLOS defines a study's minimal data set as the underlying data used to reach the conclusions drawn in the manuscript and any additional data required to replicate the reported study findings in their entirety. All PLOS journals require that the minimal data set be made fully available. For more information about our data policy, please see <http://journals.plos.org/plosone/s/data-availability>.

"Upon re-submitting your revised manuscript, please upload your study’s minimal underlying data set as either Supporting Information files or to a stable, public repository and include the relevant URLs, DOIs, or accession numbers within your revised cover letter. For a list of acceptable repositories, please see <http://journals.plos.org/plosone/s/data-availability#loc-recommended-repositories>. Any potentially identifying patient information must be fully anonymized.

Important: If there are ethical or legal restrictions to sharing your data publicly, please explain these restrictions in detail. Please see our guidelines for more information on what we consider unacceptable restrictions to publicly sharing data: <http://journals.plos.org/plosone/s/data-availability#loc-unacceptable-data-access-restrictions>. Note that it is not acceptable for the authors to be the sole named individuals responsible for ensuring data access.

We will update your Data Availability statement to reflect the information you provide in your cover letter.

>>> I now made the data freely available in the Open Science Framework. It can be accessed via the link in the data availability statement: *The data that support the findings of this study are openly available in the Open Science Framework: Bartels, S. L. (2022, October 5). “Impact of the COVID-19 Pandemic on Early Career Dementia Researchers: A Global Online Survey”. Retrieved from* [www.osf.io/fr8j2](http://www.osf.io/fr8j2) .

7. We note that you have stated that you will provide repository information for your data at acceptance. Should your manuscript be accepted for publication, we will hold it until you provide the relevant accession numbers or DOIs necessary to access your data. If you wish to make changes to your Data Availability statement, please describe these changes in your cover letter and we will update your Data Availability statement to reflect the information you provide.

>>> The manuscript can be published as soon as it is accepted. Data can be accessed via [www.osf.io/fr8j2](http://www.osf.io/fr8j2) .

8. One of the noted authors is a group or consortium "ISTAART PIA". In addition to naming the author group, please list the individual authors and affiliations within this group in the acknowledgments section of your manuscript. Please also indicate clearly a lead author for this group along with a contact email address.

>>> We adjusted the acknowledgement section accordingly: *Furthermore, we are grateful for the input from the members of the Professional Interest Area to Elevate Early Career Researchers (PEERs PIA), namely Dr. James Quinn (Programs Chair), Dr. Naiara Demnitz (Communications Chair), Dr. Tengfei Guo (Continental Lead-Asia), and Dr. Diana Karamacoska (Continental Lead-Australia). Next to these four members, the authors SLB (Continental Lead-Europe), CES (Vice Chair), WSB (Continental Lead-South America), LAW (Continental Lead-North America), RF (Continental Lead-Africa), and AS (Chair) were part of the ISTAART PEERS PIA. CES is the current chair and questions related to the ISTAART PEERS PIA can be directed to her:* [*Beth.Shaaban@pitt.edu*](mailto:Beth.Shaaban@pitt.edu)*.*

Additional Editor Comments:

Your manuscript has been assessed by one peer-reviewer and their report is appended below. The reviewer comments that the manuscript could be strengthened by providing more detail to certain aspects of the study, including the introduction, methodology, and discussion section. In addition, the reviewer states that the results should be interpreted more cautiously, and the limitations section requires more attention.

Please note that we have only been able to secure a single reviewer to assess your manuscript. We are issuing a decision on your manuscript at this point to prevent further delays in the evaluation of your manuscript. Please be aware that the editor who handles your revised manuscript might find it necessary to invite additional reviewers to assess this work once the revised manuscript is submitted. However, we will aim to proceed on the basis of this single review if possible.

[Note: HTML markup is below. Please do not edit.]

Reviewers' comments:

Reviewer's Responses to Questions

**Comments to the Author**

1. Is the manuscript technically sound, and do the data support the conclusions?

The manuscript must describe a technically sound piece of scientific research with data that supports the conclusions. Experiments must have been conducted rigorously, with appropriate controls, replication, and sample sizes. The conclusions must be drawn appropriately based on the data presented.

Reviewer #1: Yes

2. Has the statistical analysis been performed appropriately and rigorously?

Reviewer #1: Yes

3. Have the authors made all data underlying the findings in their manuscript fully available?

The [PLOS Data policy](http://www.plosone.org/static/policies.action#sharing) requires authors to make all data underlying the findings described in their manuscript fully available without restriction, with rare exception (please refer to the Data Availability Statement in the manuscript PDF file). The data should be provided as part of the manuscript or its supporting information, or deposited to a public repository. For example, in addition to summary statistics, the data points behind means, medians and variance measures should be available. If there are restrictions on publicly sharing data—e.g. participant privacy or use of data from a third party—those must be specified.

Reviewer #1: Yes

4. Is the manuscript presented in an intelligible fashion and written in standard English?

PLOS ONE does not copyedit accepted manuscripts, so the language in submitted articles must be clear, correct, and unambiguous. Any typographical or grammatical errors should be corrected at revision, so please note any specific errors here.

Reviewer #1: Yes

5. Review Comments to the Author

Please use the space provided to explain your answers to the questions above. You may also include additional comments for the author, including concerns about dual publication, research ethics, or publication ethics. (Please upload your review as an attachment if it exceeds 20,000 characters)

Reviewer #1: This is an interesting paper worthy of publication as it focuses on the impact on early career researchers. It is well written and has some useful recommendations at the end of the paper. I have some suggestions for strengthening the paper.
Abstract- I do think the results could include the findings about conference attendance as the change to remote delivery has been one of the few positive impacts of the pandemic. The conclusion section could be re-written to provide stronger conclusion to the abstract, rather than writing a list of content try instead to focus on the main messages from the paper.

>>> Thank you for reviewing our manuscript and for providing us with your input. Your suggestions have strengthened the manuscript, and we will explain in detail how changes were made to the previous draft to improve it.

Abstract: We agree that this is an important finding and should be included in the abstract; the conclusions were also adjusted: ***Results:*** *Survey data from n=321 ECDRs from 34 countries were analyzed (67.6% women; 78.8% working in academia). Overall, 77.8% of ECDRs surveyed indicated research delays, 53.9% made project adjustments, 37.9% required additional or extended funding, and 41.8% reported a negative impact on career progression. Moreover, 19.9% felt unsupported by their institutions and employers (33% felt well supported, 42.7% somewhat supported). ECDR’s conference attendance remained the same (26.5%) or increased (More: 28.6%; a lot more: 5.6%) since the start of the pandemic. Continental differences were visible, while the impact of the pandemic did not differ greatly based on ECDRs’ sociodemographic characteristics.*

***Conclusions****: The COVID-19 pandemic had a substantial impact on ECDRs worldwide and institutions, employers, and funding bodies are urged to consider the implications and lessons-learned when working with, managing, and promoting ECDRs. Strategies related to the pandemic and general career support to improve ECDRs career progression are discussed, including social media training, digital networking, and benefits of hybrid events. Global resources specific for ECDRs are highlighted.*

Introduction (page 3) I would advise including a definition of ECR given this is the focus of the paper.

>>> Thank you for this suggestion. We added the following to the manuscript in the first paragraph (line 61-64): *There is no universal definition for ECRs due to differences in job titles and career paths across the world. Therefore, the term ECR refers here to researchers at the undergraduate, graduate, postdoctoral, and early career faculty level that identify as being at an early career stage.*

Introduction (page 3) The first paragraph could make more of why this is an important area to look at. I appreciate the need to understand the experiences of dementia ECRs, but this paper will be read by those from different disciplines.

Introduction (page 3) it wasn’t clear if all the studies you were reviewing were specifically conducted to explore the impact of the pandemic on researchers.

>>> In the introduction (second paragraph), we elaborate on the reported impact of the pandemic on ECRs from various fields (e.g., animal behaviour, neurology, autism) as we agree that this manuscript will not only be read by dementia researchers. Reasons for ECRs to be of special interest are mentioned in the first paragraph (line 58-61: “*ECRs have a less established record of independent funding or publications, deal with a more vulnerable income situation, and are therefore strongly affected by research-related and current circumstantial stressors.”* And line 87-89: *“The current increased attention on COVID-19 research projects has changed the landscape of funding bodies, offering novel opportunities for some ECRs, but can also reinforce pre-existing inequities in academia.”*

In the second paragraph (page 4), we now added the months in which these ECRs were surveyed (when the information was available). In the third paragraph (page 5), we furthermore present findings from a report published in Nature, which reviewed over 7600 ECRs across fields. Only in the fourth and fifth paragraph, we then introduce the topic dementia researcher, also introducing the early career dementia researcher (ECDR) acronym:

*Across various research fields, the impact of the pandemic on the academic and personal status of ECRs is being investigated. For instance, a survey of researchers working in Animal Behavior and Welfare, other biological sciences, or social sciences conducted in July 2020 revealed that postgraduate students, research associates, and non-permanent jobholders were more likely to worry about their future compared to permanent jobholders (4). Amongst a group of Italian neurology trainees, 60% of those surveyed in April 2020 indicated a reduction in research activities, and about 70% fear the negative impact of the pandemic on their training (5). In the same month, trainees and early career faculty members of the Association for Academic Surgery and the Society of University Surgeons reported a greater negative impact of the pandemic on productivity, and having received less guidance from their institution compared to advanced career faculty counterparts (6). Furthermore, a survey of the Autism Science Foundation and Autism Speaks presents that 85% of surveyed ECRs experienced reduced productivity, particularly struggling with recruitment of research participants, and dealing with increased needs at home and personal mental health (7).*

*The COVID-19 pandemic and impact may also have lasting consequences, on ECRs’ career progression (8). A report published in Nature presents results from over 7600 multi-disciplinary postdoctoral researchers worldwide, highlighting that 61% of respondents worry about the pandemic’s negative effects on their career prospects (9). ECRs in cardiovascular research fear reduced opportunities for training, peer learning, networking, conference presentations, and recruitment issues impacting publications of original research, which may affect their career progression (8). The current increased attention on COVID-19 research projects has changed the landscape of funding bodies, offering novel opportunities for some ECRs, but can also reinforce pre-existing inequities in academia (10).*

*In dementia research specifically, important progress, for instance in blood-based biomarkers for early detection and prevention of Alzheimer’s disease, was achieved despite the pandemic (11). Research conducted in 2020 and 2021 also investigated the impact of the pandemic on people with dementia and their carers, highlighting severe disruptions of routines that support mental and physical health in these population (12, 13). Dementia is recognized by the World Health Organization as a public health priority and research is promoted as one of the actions in the Global action plan on the public health response to dementia 2017-2025 (14). However, investments in the COVID-19 illness are thought to be higher than those dedicated to dementia research and treatment (15), creating not only an issue for people affected by dementia, but also ECRs and their dementia-related work.*

Introduction (page 4) you discuss important studies that were conducted despite the pandemic. Would be worth a reference to those studies that were conducted to explore the impact of the pandemic on people with dementia and carers, these happened despite all the challenges around recruitment.

We agree that it is very relevant to also highlight people with dementia and their carers, and the research that targeted them specifically in 2020/2021. We therefore added the sentences above (last paragraph) to highlight more dementia-specific research.

Method (page 6) please provide more details of the questionnaire topics, this could be in the form of a supplementary document.

>>> The information was added to the methods section. Furthermore, the not peer-reviewed report can be accessed via the link in the references. Survey section (page 7): “*The survey created in English using www.surveymonkey.com, and covered several topics (e.g., experiences, job and workplace, conference attendance, publishing, moving countries, leaving academia), including the impact of COVID-19. The full survey report (non-peer reviewed) with descriptive information can be accessed elsewhere (17).”*

Method (page 6) it would be useful to contextualise the time period data collection period in relation to the pandemic

>>> Thank you for this suggestion. As this was a global survey, we decided to not add how countries reacted at this point in time, as the approaches varied greatly. Therefore, we added the following general information (page 7): “*The survey was distributed via social media, newsletters, podcasts, blogs, e-mails to ISTAART ECDRs, various departments, institutions, networks and charities, and was available between the 1^st^ of September, 2021, and 31^st^ of October, 2021 (towards the end of the third COVID-19 wave according to the World Health Organization).”*

Results (page 9) “delay I their projects” is that their own projects (e.g. PhD project) or the PIs project? Or is that not clear in the data. Delays have different implications depending on roles within the project.

>>>Unfortunately, this information is not available through our questionnaire, which was now included as a limitation, as we agree that the implications can vary. Limitations (page 20): *Also, it was not assessed how the delayed ECDRs projects were funded; this information would have been useful to interpret the described impact in more detail.*

Results (page 9/10) I would advise I more cautious interpretation of the findings. You state nearly two-thirds felt at least somewhat unsupported but the data shows only 19.9% felt unsupported. Whilst, 41.2% felt employers were generally supported but could do better, I don’t see this data as implying they were somewhat unsupported because they said they were generally supported. I would apply this comment to how you have reported the results in the abstract and on page 13 of the discussion section where you say only 1 in 3 ECRs were satisfied with the support.

>>> Thank you for highlighting that this phrasing was misleading. We now adjusted the way we reported the results in the abstract and discussion section to improve clarity.

Abstract (results): *Moreover, 19.9% felt unsupported by their institutions and employers (33% felt well supported, 42.7% somewhat supported). ECDR’s conference attendance remained the same (26.5%) or increased (More: 28.6%; a lot more: 5.6%) since the start of the pandemic. Continental differences were visible, while the impact of the pandemic did not differ greatly based on ECDRs’ sociodemographic characteristics.*

Results section (page 10/11): “*Among respondents, 33% found that their institutions or employers supported them well during the pandemic, while 42.7% indicated that their institution or employers were generally supportive but could have been better, and 19.9% felt unsupported by their institution or employers. Also, 34.2% (28.6% “more”, 5.6% “a lot more”) of participants reported increased conference attendance, while conference attendance was the same for 26.5% of the surveyed ECDRs since the start of the pandemic. Further details can be found in Fig 1 (a.-f.).”*

Discussion (page 14)*: Only 1 in 3 ECDRs surveyed was fully satisfied with their institution’s/ employer’s support, while the majority expressed receiving either no support, or that institutions/employers was supportive, but could have done more. While the pandemic strained academics of all career stages, this expressed feeling of neglect underlines the necessity for the research community to increase efforts and support for ECDRs, as also highlighted in other fields (7, 18, 19).*

Discussion (page 13) with the discussion of the gender differences in the data it might be worth a reference to the “dementia research career pipeline” 2022 paper which looked at gender differences.

>>>Thank you for highlighting this manuscript, indeed a useful and relevant reference to discuss in our article as well. The discussion was adjusted accordingly (page 14): *No differences related to ECDRs’ gender (men vs. women) were found in the present sample, and previous studies on the impact of the COVID-19 pandemic in relation to gender are inconclusive (4, 21, 22). Evidence exists that women in dementia research get published less, receive less funding, and transition into advanced academic positions at disproportionally lower rates than men (20) and gender disparities for ECDRs require more attention prospectively.*

Discussion- before you get to discussing the lessons learnt it is worth some discussion of the finding about conference attendance.

>>> The mentioned “dementia research career pipeline” was a helpful reference for us in this regard as well. We adjusted the abstract (as described above), as well as in the discussion section (first paragraph, page 14): *A positive impact of the COVID-19 pandemic was the reported increase in conference attendance in ECDRs. Hybrid and online events enabled broader audiences to attend dementia-related events, which was also highlighted in other reports (20), and this topic is discussed further below.*

Discussion section 3: ***Attending and presenting at events.*** *Most in-person events were cancelled or postponed during the pandemic, robbing ECDRs of the exposure to and experience of presenting their work to a live audience and receiving direct feedback from fellow investigators. On one hand, digital conferences may limit direct interactions. On the other hand, employers should still encourage professional society online presentations and participation in online conferences to allow ECDRs to receive feedback and practice their communication skills. The present findings show that conference attendance generally increased in ECDRs since the start the pandemic, which can be seen as a positive impact. ECDRs can also practice presenting at digital/ hybrid research group meetings of their own or collaborating institutions. Presentations can be recorded and uploaded to institutional or personal websites to facilitate dissemination further and demonstrate ECDRs’ expertise. ECDRs are encouraged to provide feedback to fellow researchers during digital events, as even a tweet or message in a chat can increase one’s network. Furthermore, participating in online conferences is always substantially less expensive than travelling to in-person events, which may explain the reported increase in conference attendance by European, Asian, and South/Central American ECDRs. In 2020, more women attended large dementia conferences (20), and online events might help ECDRs with children or other care or household responsibilities to combine work with personal tasks. Moving forward, hybrid events are likely more inclusive for ECDRs and facilitate international participation, specifically when project and training budgets are small. Online conference formats are thought to enhance sustainability, equity, and inclusivity (25) and the dementia field can benefit from this approach.*

Discussion (page 14) you mention continental trends with regards to reactions to the pandemic, worth considering issues around access to technologies both for researchers and participants e.g. for remote interviewing.

>>> Technology availability and use is indeed a perspective of relevance to the manuscript. We therefore added the following to the discussion section (page 15): *Continental trends, although not statistically analysed due to small sample sizes, revealed that the impact of the pandemic on ECDRs varied around the world. This observation could be explained by the governmental and cultural reactions to the pandemic, which differed between countries. Furthermore, access to technologies to switch from face-to-face to remote data collection methods might not have been available to all ECDRs or participants, leading to negative consequences in some contexts. In the U.S., for instance, smartphone ownership increased in older adults (65+ years of age) from 46% in 2018 to 61% in 2021 (24), which is a positive direction for remote dementia research although this ability to participate remotely does not appear available to all older adults.*

Discussion (Page 15) Data collection and publishing, I think there are other ways for ECRs getting findings out there and building their profile e.g. through twitter or tik tok.

>>> Thank you for this comment. While we agree, that twitter and other social media platforms are important to disseminate results, most universities still assess the number of peer-reviewed articles published first. The societal impact, which might certainly be enhanced through social media activities, is a criterion for career progression at some universities, but not all. We added these thoughts to the discussion (“Data collection and publishing”, page 16/17). “*”Moreover, social media outlets, including Twitter, Facebook, LinkedIn, TikTok, or Instagram, represent ways to disseminate research findings. ECDRs can either share hyperlinks to publications or highlight preliminary results via these platforms to reach a wide audience, which can have an additional societal impact. There is an academic movement towards tracking this type of impact via Altmetrics (26).”*

Discussion (Page 16)- A couple of issues to consider for funding. One for funders providing funding for longer periods e.g. UK Alz Soc recently changed to funding 4 years of a PhD as recognised students do need longer than 3 years. Second funding to be sufficient for people to be paid living wages e.g. with the current crisis PhD stipends are not sufficient and many PhD students are taking on second jobs.

>>> This is a great example in which funding was adjusted positively. We therefore added it to the manuscript. Discussion 5. Funding (page 18): “*One funder (the UK Alzheimer’s Society) has proven that adjustments of ECDR contracts are possible, as they extended fellowships to four years during the pandemic, and other funders should follow this example. Moreover, fellowships and contracts should provide sufficient funds for ECDRs to cover living wages, which is currently not always the case. It is not uncommon for students to have a second job to cover their costs, potentially leading to increased stress and time restrictions for conducting dementia research.”*

Discussion (Page 17), promotion, again this is just my opinion, but one thing is to include Covid-19 impact statements in promotion applications so people can talk about the impact on them e.g. if they had to reduce hours for caring duties during the pandemic.

>>> We agree that this is a helpful s suggestion and therefore promote it in our manuscript to support ECDRs with a practical tip. Discussion 6. Promotion and tenure review requirements (page 19): *Finally, ECDRs themselves can consider including a “COVID-19 impact statement” in promotion applications, in which the individual impact is highlighted, such as reduced working hours due to caring duties during the pandemic or issues with recruitment due to lock-down.*

Discussion (Page 18)- improve ECRs well-being. Support for ECRs should also include support for line managers and PIs, emotionally supporting ECRs can take its toll.

>>> Proving support/ help/ care can indeed be challenging in itself and we added this remark in the discussion section 7. Improve ECDRs well-being (page 19): *Supervisors, mentors, or managers should generally check-in on ECDRs, and stress-management courses can also be offered online. As it can be challenging to support an ECDR, particularly in times of crisis, supervisors and anyone providing help might in turn benefit from emotional guidance themselves. Being aware of one’s mental health, engaging in coping strategies, such as exercises, meditation, mindfulness training, and sharing experiences with others may be helpful.*

Discussion (page 18) limitations. You need to acknowledge that sample self-defined as ECRs so it looks like you have some associate prof/ Profs in the sample with aren’t typically seen as ECRs. The study didn’t specifically include mid-career dementia researchers. By using questionnaire methods it meant that pertinent issues couldn’t be followed up with participants as could be done with an interview e.g. clearly some people were unable to finish their research- why was that?

>>> We adjusted the limitation section, as indeed some associate professors joined the survey and were not excluded from the analysis. The subjective experience of being early in one’s career was seen as more important in this survey as cut-off scores (e.g., 7 years of working in dementia research), which appear artificial and do not consider un-traditional career paths (e.g., working part-time or leaving and returning to research while also holding a clinical position). This survey had the aim to assess the impact of the pandemic across ECDRs quantitatively, but we agree that qualitative methods help to describe the context in more detail. Qualitative methods are very time consuming and the group of researchers did not have the resources to perform this type of study during the pandemic. We therefore added this as a suggestion to the limitations section as it will be helpful to advance support for ECDRs. Limitations (page 20/21): *Study limitations include an over-representation of participants from Central Europe (44.2%) and North America (30.8%), while only a small proportion of participants were based in Asia, Africa, Australia, South America, or South-East European countries. Although several channels were used to engage ECDRs around the world, recruitment bias is likely to still be present. Furthermore, participants self-identified as being in their early career, but associate/ full professors (4%) also joined this survey. Some countries might view these titles as mid- or advanced-stage research positions, potentially introducing a bias in the results. In the present study, the subjective feeling of being at an early career stage was of primary interest. If differences based on objective career stage and title rather than self-identified career stage are of interest in future work, data from such respondent could be removed from analysis or years of experience working in dementia research could be included as a control variable in multivariable modeling. Also, it was not assessed how the delayed ECDRs projects were funded; this information would have been useful to interpret the described impact in more detail. Finally, a more advanced statistical analysis was not suitable due to sample heterogeneity. Prospectively, additional lifestyle and personal factors could be explored, qualitative information might help to understand the individual context better, and a comparison to non-pandemic challenges would be interesting.*
